# Dental Notes

**Published:** 1899-12

**Authors:** William Rollins

**Affiliations:** Boston, Mass.


### DENTAL NOTES.

BY WILLIAM ROLLINS, BOSTON, MASS.

### NOTE IV. A FILE- AND SAW-HOLDER.

I HAVE always believed in contours, and have tried to make the finishing of such fillings as rapidly as possible. One of the instruments designed for this is here shown. In an earlier form it was figured in this journal some years ago. The improvements consist in more rapid means of adjusting the files and saws. It differs from other frames in principle. These hold the saw by tension, therefore with them it is not practical to have the instruments in a curved form. This holds them firmly at each end. This entire independence of the ends permits the saws or files to take a curve as shown in the plate, where this is demonstrated in section and perspective. The gum-guard prevents the file or saw from plunging into the gum; this is a comfort in finishing the neck of the filling. The blades are held by a hardened steel nut at each end. These nuts have knife edges which, making little cuts in the edges, hold securely. Flexible files fitted to this holder are very convenient means of finishing fillings.

